# Evaluation and Management of Sudden Death Risk in Repaired Tetralogy of Fallot

**DOI:** 10.3390/jpm13121715

**Published:** 2023-12-15

**Authors:** Eiad Habib, Komandoor Srivasthan, Hicham El Masry

**Affiliations:** 1 Division of Internal Medicine, Mayo Clinic, Scottsdale, AZ 85259, USA; habib.eiad@mayo.edu; 2 Division of Cardiology, Mayo Clinic, Scottsdale, AZ 85259, USA; srivathsan.komandoor@mayo.edu

**Keywords:** sudden cardiac death, risk stratification, ventricular tachycardia, tetralogy of Fallot, arrhythmia

## Abstract

Although substantial progress has been made to prevent sudden cardiac death in repaired tetralogy of Fallot patients, ventricular arrhythmia and sudden death continue to be major causes of morbidity and mortality in these patients. Greater survival in contemporary cohorts has been attributed to enhanced surgical techniques, more effective management of heart failure, and increased efforts in risk stratification and management of ventricular arrhythmias. More recently, our understanding of predictive risk factors has evolved into personalized risk prediction tools that rely on comprehensive demographic, imaging, functional, and electrophysiological data. However, the universal applicability of these different scoring systems is limited due to differences between study cohorts, types of anatomic repair, imaging modalities, and disease complexity. Noninvasive risk stratification is critical to identify those who may derive benefit from catheter ablation or cardioverter defibrillator implantation for primary prevention. Ultimately, assessment and risk stratification by a multidisciplinary team is crucial to analyze the various complex factors for every individual patient and discuss further options with patients and their families.

## 1. Introduction

Tetralogy of Fallot (TOF) is the most common cyanotic congenital heart defect [1]. Since the first surgical repair of TOF over 65 years ago, significant advancements in its management have led to increased survival. Advances in surgical interventions combined with enhanced imaging techniques, catheter-based and electrophysiology procedures, heart failure management, and improved surveillance have all contributed to significant progress in long-term outcomes [1]. However, late mortality from sudden cardiac death (SCD) was recognized in the 1970s and has continued to be an area of concern since then [2]. The principal etiologies presumed to contribute to SCD are ventricular arrhythmia and heart failure, though other mechanisms may be involved [3]. Although significant improvement in the detection, treatment, and prevention of such triggers has occurred, SCD remains a leading cause of mortality in this population [4]. Historical cohorts reported up to an 8.3% mortality rate for patients over 35 years [5] (which is 20 times higher than that of the general population); more recent cohorts are showing an annual mortality rate of 1% [6]. As a result, in these patients, we have been observing the constant evolution of approaches to enhance the prediction and management of arrhythmia substrates. Efforts have been made to develop comprehensive scoring systems that identify patients at high-risk of SCD and guide the decision for defibrillator implantation [7,8,9].

This article aims to explore etiologies of SCD in TOF patients, review the current literature exploring risk stratification for SCD, and examine the role of different interventions to reduce this risk.

## 2. Mechanisms of Ventricular Tachycardia in Repaired TOF

**Figure 1:**
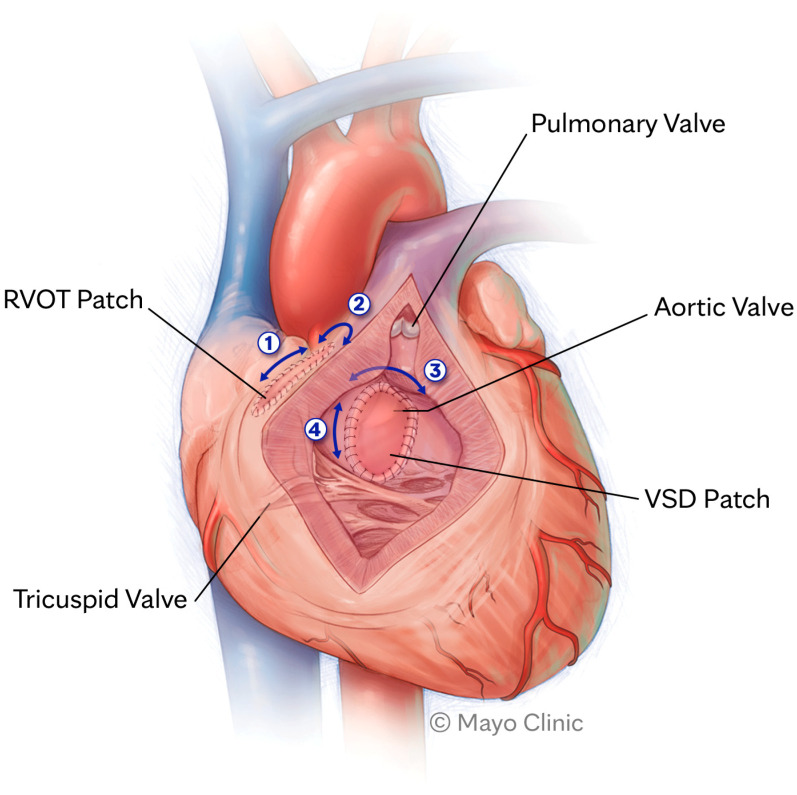
Anatomic isthmuses that may trigger ventricular tachycardia. RVOT, right ventricular outflow tract; VSD, ventricular septal defect.

The incidence of ventricular arrythmias in adults with TOF is unknown, but it is likely the dominant mechanism of SCD in this patient population [10]. The leading form of ventricular arrhythmia is monomorphic ventricular tachycardia (VT). A large study evaluating the effectiveness of implantable cardioverter defibrillators (ICDs) for primary and secondary prevention in repaired TOF found that >80% of ICD therapies were targeted specifically at monomorphic VT [11]. Such VTs are usually fast and not well tolerated [12]. Similar to VT related to structural heart disease (including ischemic cardiomyopathy), the leading mechanism of arrhythmia is macro re-entry. The substrate for re-entry in patients with repaired TOF depends on critical anatomic isthmuses (AIs) confined between areas of myocardial fibrosis or surgical patch and anatomic barriers, most commonly the pulmonic or tricuspid annulus [10]. Conduction between those barriers can slow down over time, facilitating re-entry around fixed barriers, most commonly between the ventricular septal defect (VSD) patch or right ventricular outflow tract (RVOT) incision and the pulmonic or tricuspid valve annulus [12,13]. Figure 1 highlights the AIs that may trigger VT. Both presence and geometry of AIs are influenced by variations in original anatomy, type of surgical repair, and presence of concurrent fibrosis [10].

Slow conduction (defined as conduction velocity < 0.5 m/s) across any pathway of interstitial fibrosis surrounded by inexcitable borders is shown to be a sensitive and specific marker for the presence of an arrhythmogenic isthmus that can sustain VT. Detailed electroanatomic mapping studies have demonstrated that lack of any slowly conducting anatomic isthmus in the RVOT of such patients correlates with lack of VT inducibility during electrophysiologic provocation, and absence of clinical VT on 2-year follow-up. On the other hand, the presence of a slowly conducting AI correlates with VT inducibility in 93% of patients [13].

Thus, the pathogenesis of VT in repaired TOF is related to an initial substrate due to the congenital anomaly and subsequent surgical corrections. Persistent hemodynamic insults, including volume/pressure overload, dilation, hypertrophy, and aging, lead to progressive fibrosis and slow conduction [10]. Triggers (such as PVCs and non-sustained ventricular tachycardia (NSVT)) that are facilitated by diffuse fibrosis coupled with susceptible substrate allow the generation of sustained VT [10].

In a minority of patients, VT substrates may be remote from the defined AIs, for instance, in relation to the duration of right ventricular ischemia during surgery or postcardiotomy fibrosis in others, and may create substrate changes in non-RVOT locations [10]. These patients are also at an increased risk of bundle branch re-entrant VT, especially with underlying conduction abnormalities and commonly present with right bundle-branch block [13]. Polymorphic VT or ventricular fibrillation are more frequently encountered in patients with suboptimal hemodynamics and worsening ventricular function. The relationship between these arrhythmias and the AIs remains unclear.

### Bradyarrhythmia and SCD in Repaired TOF

While most sudden deaths in repaired TOF patients are assumed to be related to ventricular tachyarrhythmias and heart failure, a strong association between late-onset high-grade atrioventricular (AV) block and SCD exists and is well documented in the literature [14,15]. A large epidemiologic analysis noted an incidence of 1% in this population, with 0.6% undergoing permanent pacemaker implantation [16]. Patients typically present with syncope or cardiac arrest, often in the absence of a known trigger or electrocardiogram (ECG) changes [17]. As with degenerative conduction disease, AV block risk factors include a prolonged PR interval, left fascicular disease, and bifascicular block [18]. Additionally, delayed recovery of postoperative AV block following initial surgical repair (occurring beyond the third day) has been independently linked to a six-fold increase in risk of SCD over a 30-year follow-up [18]. However, it is important to note that these results pertain to a cohort who underwent a historic surgical repair approach, and various other risk factors may have contributed to their risk for SCD. To date, no prospective studies have examined the role of screening strategies in risk stratification for late AV block in repaired TOF patients. Whether extended invasive or noninvasive rhythm monitoring can detect occult conduction disease before SCD is yet to be seen. Finally, the utility of invasive electrophysiology evaluation to assess conduction intervals and, ultimately, its role in risk stratification of these patients remains unclear [19].

## 3. Risk Stratification

### 3.1. Risk Factors

Although SCD is among the leading causes of mortality in adults with TOF, recent studies have shown that the annual risk of SCD in adult patients with repaired TOF is 0.2% [20]. Since this number is far too low to warrant invasive risk stratification or implantation of ICDs for every patient, significant interest has been shown in identifying individuals at higher risk for SCD. To date, no single risk factor has sufficiently stratified the risk of SCD in repaired TOF patients. However, various risk factors allow for a more precise prediction of risk within this population [5]. Early multicenter studies demonstrated that the most common risk factors for SCD were left or right ventricular (RV) systolic dysfunction, prolonged QRS duration (≥180 milliseconds), late age of repair, and transannular patch repair [5]. A recently published meta-analysis of over 7000 patients with repaired TOF further noted the consistent impact of age, QRS duration, older age of repair, previous palliative shunts, atrial arrhythmias, and RV or left ventricular (LV) dysfunction on the risk of SCD in these patients [21].

### 3.2. Age

Older patient age, late age of initial repair, and late age at pulmonary valve replacement (PVR) have all been linked to an increased risk of ventricular arrhythmia in TOF patients. A large multicenter observational study noted age at initial repair to be an independent predictor of VT in univariate analyses, in addition to being a predictor of SCD in multivariate analyses [5]. Multiple prospective and retrospective multicenter registries demonstrated an association between older age at PVR and risk of VT, SCD or appropriate ICD therapies [21,22]. Yet, examining the influence of age on surgical repair independently from the surgical technique is challenging, as advancements in surgical techniques have occurred simultaneously with the push for early surgical repair. Patients undergoing repair at an older age are more likely to have increased myocardial fibrosis in light of multiple surgical interventions (hence surgical scars), worsened hemodynamics, and increased RV remodeling and hypertrophy.

### 3.3. Clinical Risk Factors

The presence of arrhythmia-like symptoms, including palpitations, pre-syncope, or syncope, has been linked with a higher incidence of malignant arrhythmias and SCD [23,24]. Khairy et al. [25] demonstrated that a reported history of syncope prior to the electrophysiological study was predictive of a higher risk of inducible monomorphic or polymorphic sustained VT (OR 4.9, *p*-value < 0.0001). Moreover, heart failure symptoms, including dyspnea, orthopnea, and edema (particularly NYHA class II or higher), have been independently associated with higher rates of SCD and ventricular arrhythmia [9,24,26].

Cardiopulmonary exercise testing is typically utilized to assess the extent of exercise intolerance in congenital heart disease (CHD) patients. A study by Müller et al. [27] revealed that predicted peak oxygen uptake (VO_2_) that was ≤65% or ventilatory efficiency (expressed as V E/V CO_2_ slope) ≥31 were independent predictors of sustained VT and mortality. A similar trend was observed in another prospective trial, demonstrating that a peak VO_2_ ≤ 17 mL/kg/m^2^ was associated with ventricular arrhythmias and all-cause mortality [9].

### 3.4. Surgical Risk Factors

As mentioned above, one of the proposed risk factors for SCD in repaired TOF patients is the effect of the surgical era in which the repairs occurred. Mechanisms for SCD that have been postulated in such patients include prolonged cyanosis and extensive right ventriculostomy, which have been shown to increase the incidence of ventricular arrhythmias [28]. The location and extent of ventriculotomy in these patients influence the dimensions of an AI that can propagate VT. A multicenter analysis indicated that the increasing complexity of defects in repaired TOF patients independently confers a higher risk of SCD [7]. Complex repairs, including patients with pulmonary atresia or a double outlet RV, resulted in a four-fold increased risk of SCD compared with conventional anatomy [7]. Another multicenter cohort study demonstrated that patients who underwent repair with a conduit associated with RV remodeling/hypertrophy had a higher mortality rate than patients with native outflow tracts [29]. In addition, palliative shunts before definite repair have been associated with ventricular arrhythmias [23]. Conversely, TOF patients who undergo transannular patch repair or who have an intact ventricular septum, even with significant RVOT obstruction, are at low risk of VT and SCD. In these cases, the repair circumvents the creation of an AI [23].

### 3.5. QRS Duration

Most patients with repaired TOF have a complete RBBB. Additional hemodynamic alterations (such as increased right ventricular pressure or volume overload) can induce ventricular fibrosis, further slowing down conduction and causing prolongation of the QRS complex [30]. A landmark study identified a strong association between QRS duration of ≥180 milliseconds and VT/SCD [5]. However, this older study examined patients from a different surgical era, while contemporary surgical techniques correlate with narrower QRS durations. As such, QRS duration must be evaluated within the context of the surgical era, and in current populations, a lower QRS duration (150 milliseconds) may be sufficient to indicate an AI and risk of VT [30]. More recent cohorts have confirmed these associations and noted that the risk association is continuous instead of dichotomous [23,31].

### 3.6. QRS Fragmentation

Fragmentation of the QRS complex (fQRS) is defined by the presence of three or more notches of the widened QRS in two or more contiguous leads. It has been associated with myocardial fibrosis and increased risk of SCD [32,33]. fQRS is present in up to 40% of patients with repaired TOF [34,35], and is more commonly found in patients with RV dysfunction, dyssynchrony, or fibrosis [34,36]. A prospective, multicenter study evaluated the influence of fQRS on mortality in patients with repaired TOF. The extent of fQRS was superior in predicting mortality compared to QRS duration, and fQRS was also predictive of ventricular arrythmias [34]. Another long-term follow-up study of repaired TOF patients who had implanted ICDs noted that QRS fragmentation was the sole independent predictor of appropriate ICD therapies in patients with devices inserted for primary prevention [37].

### 3.7. Cardiac Imaging

Echocardiography and cardiovascular magnetic resonance (CMR) can identify subclinical features that can recognize TOF patients at increased risk for SCD. Multiple studies have shown an association between RV remodeling (RV size and hypertrophy) and ventricular arrhythmias [29,38,39]. In addition, ventricular dyssynchrony and abnormalities in global longitudinal strain are implicated [40,41,42]. The presence and extent of late gadolinium enhancement (LGE) on CMR have been associated with spontaneous and inducible VT [39,43]. A study by Khairy et al. [11] demonstrated that elevated LV diastolic pressures were associated with a higher risk of ventricular arrhythmias. However, the thresholds for LV/RV dysfunction that translate to a significant risk of VT/SCD remain unknown.

### 3.8. Genetic Syndromes

Patients with genetic syndromes, especially DiGeorge syndrome (22q11 microdeletion syndrome) associated with pulmonary atresia and repaired TOF, have a higher mortality rate and as much as five times higher risk of SCD [44]. However, these patients have higher rates of LV systolic dysfunction and more deleterious effects resulting from extra-cardiac lesions that may be confounding factors for higher rates of mortality compared to TOF patients without genetic syndromes [45].

Table 1 summarizes the anatomical, clinical and EP risk factors for SCD in this population.

## 4. Risk Scores

### 4.1. Khairy Score

In a landmark analysis published in 2004 [25], Khairy et al. showed that inducible monomorphic or polymorphic VT following rigorous programmed ventricular stimulation increased the likelihood of SCD five-fold compared to established noninvasive risk factors alone. Conversely, noninducibility was shown to have a favorable prognosis with 89% 15-year survival. Extrapolating from results, the Khairy score [11] was published in 2008 to better predict the need for ICD implantation for primary prevention in this patient population. Although widespread adoption and implementation of this risk score have provided significant benefits, clinicians should be aware of some important pitfalls when using this score in contemporary management. Firstly, calculating the score requires the derivation of LV end-diastolic pressure in addition to results of an invasive electrophysiology study (EPS). This limits its usefulness as a screening tool. An approach that is commonly used is to refer patients with multiple noninvasive risk factors to have an invasive EPS to further stratify their risk of arrhythmia and SCD [10]. Secondly, patients with complex defects (such as pulmonary atresia, double outlet RV, or Rastelli repair) were excluded from the study cohorts, which may underestimate the risk of SCD within these specific groups of patients [7]. Finally, patients included in the initial cohort had multiple comorbidities by contemporary standards, including a late age of repair (average age 4.5 years), 50% requiring palliative shunts before definitive repair, 17% with documented sustained VT, and 25% with a history of syncope [25]. A reanalysis of the Khairy score that was recently published highlighted a suboptimal C-index of 0.6 in predicting SCD [50]. As discussed previously, it is plausible that older surgical techniques, along with longer durations of cyanotic circulation during childhood, may have resulted in more significant myocardial fibrosis. This, in turn, lead to a larger substrate for ventricular arrhythmia and a greater risk of SCD that may not be appreciated within a contemporary cohort.

To enhance the versatility of the Khairy score while recognizing the robust and comprehensive disease discrimination that CMR can provide in patients with repaired TOF, Bokma and colleagues devised a model for predicting mortality and ventricular arrhythmia in 2017 [51]. This model utilized the noninvasive variables of the Khairy score and supplemented them with CMR-derived metrics related to significant systolic dysfunction (LVEF < 45% and RVEF < 30%), translating to an augmented C-index of 0.75.

### 4.2. Contemporary Risk Scores

More recently, four large multicenter analyses have formulated risk scores with greater sensitivity and specificity than the Khairy score for prediction of SCD in TOF patients. The Spanish ACHD network [7] evaluated over 3500 patients with a wide range of congenital heart diseases, including 360 patients with repaired TOF, to create a risk score with high sensitivity (C-index of 0.91) without marked reduction in specificity. Similarly, the PREVENTION-ACHD risk score [8] was devised to recognize high-risk features for SCD in congenital heart disease patients, and their cohort included 138 patients with repaired TOF. Both of these contemporary risk scores highlighted ischemic heart disease as a novel and significant risk factor for SCD, with a four- to eight-fold increase in odds [7,8]. Unfortunately, both of these risk scores were derived from all CHD patients. Although they provide high sensitivity and specificity for SCD across various CHD lesions, their predictive value, specifically within TOF patients, remains unknown.

A recently published prospective study by Ghonim et al. [9] included a large cohort of 550 patients with repaired TOF and incorporated detailed CMR and LGE burden to construct a score that exhibited a strong predictive capability for SCD risk. The score, consisting of eight risk factors, heavily relies on CMR measurements (68/100 points) and more specifically on the extent of RV LGE (40/100 points). Patients in the highest-risk group (≥51 points) had a 4.4% annual mortality rate and 36% mortality at ten years, while those in the low-risk group (0–20 points) had <0.2 yearly mortality and 1% 10-year mortality [9]. It is worth noting that after accounting for the degree of LGE, characteristics of surgical repair, QRS duration, and NSVT were not independently predictive of outcomes. This further validates the complex interplay between such parameters as surrogate indicators of myocardial fibrosis [52]. An important caveat is that accurate quantification of RV LGE requires a high level of expertise to guarantee reproducibility. Given how significant this value is to determining the overall score, each institution must consider its ability to accurately provide CMR metrics to avoid inappropriate implementation to their specific patient population [9].

Another study published earlier this year [53] used a machine learning algorithm that incorporated 57 variables from electronic records of patients with repaired TOF to develop a scoring system that could predict a composite outcome of mortality, resuscitated sudden death, heart failure admissions, and sustained VT. Once developed, a refined model that included the ten strongest risk factors (labeled “AiTOR”) performed well in an independent validation cohort with a C-index of 0.82. However, LGE data were only available for a small subset of patients (4%), which may reflect routine clinical practice and thus was not sufficiently powered to be included in the final model. Importantly, while this model could predict a composite of outcomes, very few sustained VTs or sudden deaths were noted, limiting the score’s utility in predicting VT and ICD.

As with any high-risk cohort, it is important to highlight that these risk scores do not encompass every patient who develops SCD. Studies have shown that even with a sensitivity of ~95%, low-risk patients continue to have an approximately 0.2% annual risk of ventricular arrhythmias and SCD [9]. It is yet uncertain whether complimentary strategies, such as continuous monitoring through wearable biosensors [53], contemporary multidisciplinary clinical surveillance techniques, and early intervention for structural and electrophysiological abnormalities, effectively reduce the remaining risk.

Table 2 summarizes the various tools available for risk stratification of SCD in repaired TOF patients.

### 4.3. Invasive Risk Stratification

In patients who exhibit multiple noninvasive risk factors for SCD, programmed ventricular stimulation (PVS) can be utilized as a tool for further risk stratification. Khairy et al. [25] analyzed the utility of PVS in predicting sustained VT or SCD in patients with repaired TOF. Clinical VT and/or SCD occurred in 25% of patients, although the majority had isolated VT without SCD and did not require resuscitation. A positive PVS showed a likelihood ratio of 3.8 for VT or SCD, while inducible sustained VT had a relative risk of 4.7 for VT or SCD [25]. A recently published prospective cohort study evaluated the yield of a PVS pre-pulmonary valve replacement (PVR) [54]. In total, 49% of patients had inducible sustained VT prior to PVR, and most patients underwent surgical cryoablation. In addition, 50% of patients who had surgical ablation remained inducible at postoperative EPS and subsequently underwent ICD implantation. Although this study was provocative, it was limited by inconsistency in the approach to surgical ablation. Furthermore, it is uncertain whether an analysis conducted when patients are most hemodynamically vulnerable (to the point when PVR is necessary) can accurately predict long-term arrhythmic risk following the predicted ventricular remodeling after PVR [10].

Current guidelines from the 2018 American Heart Association/American College of Cardiology Adult Congenital Heart Disease recommend invasive EPS with PVS for repaired TOF patients and further risk factors for SCD, including NSVT, QRS duration ≥ 180 milliseconds, LV systolic or diastolic dysfunction, or extensive RV fibrosis on CMR [55].

## 5. Reducing SCD Risk

### 5.1. Medical Therapy for Heart Failure

With an aging TOF population, heart failure is becoming a progressively more recognized contributor to SCD in repaired TOF patients. Causes are multifactorial and closely resemble risk factors highlighted in the scoring systems described above, including increasing age, atrial tachyarrhythmia, LV dysfunction, and valvular disease [56]. The utility of conventional medical therapy such as beta-blockers, renin–angiotensin inhibitors, SGLT-2 inhibitors, and cardiac resynchronization therapy for reducing mortality and SCD in repaired TOF patients with heart failure remains uncertain. However, these interventions may be useful in patients exhibiting LV dysfunction [57,58,59,60].

### 5.2. Antiarrhythmic Drugs

In recent decades, the predominant emphasis on arrhythmia suppression has been directed toward ablation and devices, with comparatively less attention given to antiarrhythmic therapy. Choosing a suitable medication regimen for the management of ventricular arrhythmias in TOF patients is mostly extrapolated from VT management in adult patients without CHD [10]. Limited research has been conducted to explore the medical management of ventricular arrhythmias in CHD. A small single-center study investigated the outcomes of antiarrhythmic therapy combined with radiofrequency ablation in those with drug-refractory VT [61]. The study concluded that amiodarone and sotalol, when used in conjunction with ablation, were successful at suppressing VT. The lack of specific studies on the effectiveness of various antiarrhythmics in the management of VT has resulted in inconsistent use of these drugs. Patients who present with VA, particularly polymorphic VT that is not amenable to ablation, may derive benefit from beta blockade. If ineffective, consideration may be given to a class III antiarrhythmic.

### 5.3. Implantable Cardiac Defibrillators

Patients with a history of resuscitated SCD or hemodynamically unstable VT should be offered a secondary prevention ICD to prevent SCD [10]. Guidelines from the 2018 American Heart Association/American College of Cardiology offer a class IIa recommendation for ICD implantation for the primary prevention of SCD in patients with repaired TOF and multiple risk factors [55]. These include NSVT, QRS duration ≥ 180 milliseconds, LV dysfunction, extensive RV scarring, and inducible sustained VT at invasive EPS. However, these guidelines were established before recently published contemporary risk scores that are more likely to estimate an individual patient’s SCD risk.

The two strategies currently available for ICD implantation are transvenous and subcutaneous defibrillators. Transvenous systems offer the added benefit of anti-tachycardia pacing, which has the potential to be customized for patients with a secondary indication [62], as well as bradycardia pacing for individuals with pre-existing conduction disease. When there is no pacing indication, an attractive alternative is an entirely subcutaneous device, which preserves venous access in younger patients and may circumvent the high risk of transvenous-related lead complications [63,64]. However, loss of anti-tachycardia pacing with subcutaneous ICDs can result in more shocks when compared to transvenous systems, which was highlighted by a study showing that 59% of VT in TOF patients with ICDs was terminated successfully through anti-tachycardia pacing [37]. Limited data are available to compare long-term outcomes of subcutaneous devices, although the risk of complications in appropriately screened patients is low [65,66]. The main barrier to implantation of subcutaneous devices is ineligibility in up to 40% of patients because of T-wave oversensing in the setting of a typically wide QRS and RBBB morphology associated with repaired TOF. Right-sided screening and implantation have been shown to have higher success rates (up to 75%). Nonetheless, screening failure is strongly associated with increasing QRS duration, often observed in this cohort [67].

Patients with repaired TOF experience a high rate of appropriate ICD shocks, ranging from 5 to 10% annually, and this rate does not differ between primary or secondary indication implants [68]. In addition, these patients experience increased rates of device-related complications, with a large registry reporting a 6% complication rate within 30 days of implantation and 43% by a median follow-up of 7 years [37]. The most frequently experienced complication is inappropriate shock in 25% of patients (predominantly due to atrial arrhythmia), followed by lead failure, lead infection, and bleeding [64,69]. To mitigate the risk of inappropriate shocks in patients with atrial arrhythmias, programming of appropriate detection zones, long detection times, and algorithms for SVT discrimination are crucial [52]. Lead failure occurs in 2–9% of cases and is typically managed by replacement of the lead and concurrent lead extraction [37,64]. Device infection occurs in 2–11% of patients and includes device lead vegetation, device pocket infection, or bacteremia [37,64]. Given that one third of device infections are encountered within 30 days from generator change, scrupulous wound care and appropriate monitoring may decrease the incidence of postprocedural infections [10].

Therefore, it is strongly recommended that ICD implantation and surveillance are performed at well-equipped centers with resources and expertise to manage device-related complications and provide timely management of arrhythmia.

### 5.4. Ablation of VT

In TOF patients with ICDs implanted for SCD prevention, >80% of appropriate shocks are delivered for monomorphic VT [70,71]. ICDs can effectively terminate ventricular arrhythmias but do not prevent them and, as mentioned above, have an increased risk of complications. Despite the availability and utility of anti-tachycardia pacing, about 40% of patients still require ICD shocks to terminate primarily fast VTs [37]. Thus, fast monomorphic VT is the most frequently encountered subtype of arrhythmia in repaired TOF and contributes to increased morbidity and mortality [10]. The dominant VT substrate (mainly slowly conducting AIs) utilizes a macro re-entrant circuit that relies on distinct slow conduction pathways within the RV. A minority of patients exhibit an alternative source of VT, such as conduction bundles or focal sites within the left or right outflow tracts that are also amenable to ablation [52]. Several studies have demonstrated that radiofrequency catheter ablation reduces the risk of VA, ICD shocks, and SCD in patients with sustained VT [70,71]. Moreover, the 12 lead VT ECG was shown to be a noninvasive predictor of the involved AI: most RBBB VTs were due to clockwise activation of AI3 while most LBBB VTs were due to clockwise activation of AI3 if precordial transition was <V5 while a transition of ≥V5 was due to AI1 or AI3 involvement [72]. Slowly conducting AIs can be identified during stable rhythm and do not need VT induction [10]. As such, even in cases of hemodynamic instability, isthmus-dependent VTs may be ablated and have a low rate of VT recurrence if a conduction block is achieved across targeted isthmuses [73]. In the minority of patients who exhibit a focal VT mechanism, induction of VT and mapping remain necessary for identification. Multiple anatomic studies have demonstrated that the most commonly observed AIs are Isthmuses 1 and 3. Isthmus 3 is frequently narrow and exhibits reduced wall thickness, potentially predisposing to adverse remodeling [74]. Since Isthmus 1 (mean length 3.9 ± 1.08; thickness 1.5 ± 0.3 cm) is usually broad and more challenging to transect with catheter ablation, Isthmus 3 (mean length, 1.4 ± 0.8; thickness, 0.6 ± 0.2 cm) may be considered a preferred ablation target for this circuit [74]. Outcomes of catheter ablation are generally favorable, with success rates of 70–80% acutely [71]. When catheter ablation fails, it may be attributed to inaccessible tissue (due to overlying material such as prosthetic valves or patches), hypertrophied myocardium, or pulmonary conduits attached to the outlet septum [13]. In the fast VT cohort, however, there is a higher risk of recurrent fast VT associated with poorer outcomes and possibly increased mortality, so these patients maintain a long-term indication for ICD placement [75]. Reported complication rates of catheter ablation are low, further solidifying the role of ablation as a safe and early treatment modality [10]. In the well-tolerated VT subgroup, there is significant interest in determining whether initiating first-line VT ablation upfront could eliminate the need for an ICD [75] once satisfactory ablation endpoints, including non-inducibility, are achieved [13]. While this approach has a class IIb indication in international guidelines [55,76], comprehensive outcome data are scarce, and additional long-term monitoring and follow-up for these patients is necessary before incorporating this into routine clinical practice.

### 5.5. Pulmonary Valve Replacement

Pulmonary valve regurgitation with significant hemodynamic changes is common after repaired TOF, leading to longstanding volume overload of the RV and consequent adverse remodeling (abnormal dilatation and systolic dysfunction). These changes predispose patients to ventricular arrhythmias and SCD [5,77]. While pulmonary valve replacement (PVR) improves symptoms as well as RV parameters (resulting in positive remodeling of the RV), its impact on reducing the risk for future ventricular arrhythmias, ICD shocks, and SCD remains uncertain [78,79]. A large cohort study published in 2021 reduced the burden of appropriate ICD therapies following PVR [78]. A separate multicenter registry study did not find a significant effect on recurrent sustained VT or SCD using a propensity score-adjusted analysis [79]. However, long-term outcomes from this cohort were recently reported, demonstrating a significant reduction in sustained VT and SCD (HR 0.4) [49]. Variability in study results could be attributed, at least in part, to differences in indications for intervention and timing for PVR across cohorts. This variation may have led to different levels of RV arrhythmic burden and reversible substrate.

Although PVR typically leads to lower RV volumes and improved hemodynamics, replacing the pulmonary valve does not eliminate slowly conducting AIs as a substrate for monomorphic VT [80]. Complimenting valve replacement with ablation, under the guidance of intraoperative VT mapping, has significantly reduced the risk of spontaneous monomorphic VT following surgery [81]. Additionally, for patients at increased risk of VT or those who have inducible VT preoperatively, concomitant ablation significantly reduces the need for an ICD [82,83]. However, empiric concurrent cryoablation during PVR has a high failure rate in achieving isthmus block [54]: even though the burden of VT may be reduced, up to half of cases still exhibit inducible VT after intraoperative cryoablation. Hence, consideration for implanting an ICD remains warranted in such cases [54]. Under the guidance of pre-procedure electroanatomic mapping and potential catheter ablation, a more targeted surgical cryoablation of certain isthmuses has demonstrated superior outcomes with lower rates of ventricular arrhythmias, SCD and potential ICD implantation [82,83].

Despite conflicting data, it may be reasonable to offer certain patients undergoing pulmonary valve replacement a preoperative risk assessment with an EPS and subsequent electroanatomic-guided ablation for cases of induced VT [10,55]. Such decisions should be discussed with and endorsed by a multidisciplinary team specialized in adult CHD at an expert center.

## 6. Our Approach to Risk Stratification of Repaired TOF and Primary Prevention

**Figure 2:**
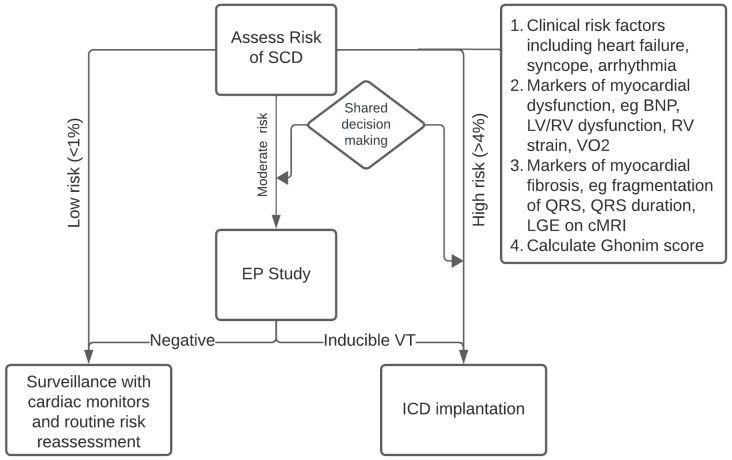
Proposed risk stratification algorithm.

As discussed in this review, risk stratification of patients with repaired TOF remains rather complex. Balancing the risk of VT-related clinical events with the risk of long-term complications after ICD implantation should be carefully reviewed with the patients and their families, and a shared decision-making model is recommended. We approach this challenge by addressing three main categories of risk factors: (1) clinical indicators, including the presence and severity of heart failure symptoms as well as any history of unexplained syncope and atrial arrhythmia; (2) markers of myocardial dysfunction, including elevated BNP, reduced RV/LV ejection fraction, RV strain and VO_2_, and (3) markers of myocardial fibrosis including fragmented QRS, QRS duration and the presence of LGE on cardiac MRI. We also calculate the risk based on the Ghonim score model to further aid in identifying the risk category. In patients with a high risk of SCD (>4%/5 years), defibrillator implantation is offered, and we typically favor transvenous ICD implantation as it offers ATP therapy and backup pacing due to the small risk of complete AV block. To reduce the risk of infection, intraoperative use of intravenous antibiotics and a pouch as well as careful postoperative follow-up are recommended. Patients at moderate risk of SCD are offered an electrophysiology study to assess for inducible sustained monomorphic or polymorphic ventricular tachycardia and ICD implantation if positive. A negative electrophysiology study has a strong negative predictive value; however, periodic re-evaluation and Holter monitors are performed for longitudinal follow-up. Patients deemed to be at low risk (<1%) are followed regularly with periodic cardiac rhythm monitors, especially for those demonstrating any LGE on cardiac MRI imaging. The value of long-term monitoring, including wearable devices and loop recorders, has not been clearly established, although it appears theoretically advantageous. Our approach to risk stratification of repaired TOF is highlighted in Figure 2.

## 7. Conclusions

SCD remains a concern in repaired TOF patients. Despite advancements in both invasive and noninvasive risk stratification, there is currently no universally accepted algorithm for identifying patients at the highest risk of ventricular arrhythmias who warrant more aggressive management. Decision-making is often difficult due to the heterogeneity of patients from various surgical eras and the variability in estimates of SCD derived from historical versus contemporary risk-scoring tools. A multidisciplinary approach involving ACHD experts as well as patients and their families is crucial to address these unique challenges. It remains uncertain whether the addition of ancillary long-term rhythm monitoring, advancements in device technology and enhanced ablation strategies will provide a more refined approach to management and alleviate the risk of sudden cardiac death in this population.

## Figures and Tables

**Table 1:** Anatomical, clinical, and EP risk factors for SCD in repaired TOF patients.

Risk Factors	Described Associations	References
**Age**	Older age Older age of Repair Older age at PVR	Atallah et al. [23], 2020, Bokma et al. [34], 2017, Gatzoulis et al. [5], 2020, Possner et al. [21], 2020, Waldmann et al. [37], 2020
**Surgical Technique**	Prior palliative shunt Ventriculotomy Transannular repair Multiple repairs	Atallah et al. [23], 2020, Gatzoulis et al. [5], 2000, Khairy et al. [11], 2008, Possner et al. [21], 2020
**Genetics**	22q11 syndrome Trisomy 21	Blais et al. [46], 2021, Kauw et al. [47], 2020, Van Mil et al. [44], 2020
**Symptoms**	Heart failure symptoms (particularly NYHA II or III) Arrhythmic symptoms (such as syncope), especially in combination with NSVT	Atallah et al. [23], 2020, Koyak et al. [48], 2013, Oliver et al. [7], 2021, Vehmeijer et al. [26], 2018
**Ischemic Heart Disease**	Coronary disease or symptomatic ischemic disease	Oliver et al. [7], 2021, Vehmeijer et al. [26], 2018
**Biomarkers**	Elevated BNP ≥ 127	Ghonim et al. [9], 2022
**Functional Assessment**	VO_2_ max < 17 mL/kg/m^2^ by cardiopulmonary exercise testing	Müller et al. [27], 2015, Ghonim et al. [9], 2022
**Ventricular Dysfunction**	LV: Abnormal systolic or diastolic function, increased LVEDP	Ghai et al. [24], 2002, Ghonim et al. [9], 2022, Khairy et al. [11], 2008, Oliver et al. [7], 2021, Possner et al. [21], 2020, Vehmeijer et al. [8], 2021
	RV: At least moderate dysfunction	Ghonim et al. [9], 2022, Oliver et al. [7], 2021, Possner et al. [21], 2020, Vehmeijer et al. [8], 2021
	Presence of LV LGE, burden of RV LGE	Ghonim et al. [9], 2022
**NSVT**	Associated with ventricular arrhythmias only when symptomatic	Bokma et al. [49], 2023, Ghonim et al. [9], 2022, Khairy et al. [11], 2008, Koyak et al. [48], 2013
**QRS Duration**	While originally stratified by QRS duration > 180 msec, contemporary cohorts suggest risk interpretation with QRS duration as a continuous variable	Bokma et al. [34], 2017, Gatzoulis et al. [5], 2000, Oliver et al. [7], 2021, Possner et al. [21], 2020
**Fragmentation of the QRS**	Presence and degree of QRS fragmentation, with the highest risk when >3 fragmented signals in 2 or more contiguous anterior leads Associated with overall mortality	Bokma et al. [34], 2017, Egbe et al. [35], 2018, Possner et al. [21], 2020, Vehmeijer et al. [26], 2018, Waldmann et al. [37], 2020

BNP, B natriuretic peptide; LGE, late gadolinium enhancement; LV, left ventricle; LVEDP, left ventricular end-diastolic pressure; NSVT, non-sustained ventricular tachycardia; NYHA, New York Heart Association; PVR, pulmonary valve replacement; RV, right ventricle.

**Table 2:** Risk stratification tools.

Risk Score	Variables	Risk Classification	Predictive Value (C-Index)
**Khairy et al. [11]**, **2008** **Bokma et al. [51]**, **2017 modifications**	*12-point risk score:* LVEDP ≥ 12 mmHg (3) NSVT (2) Ventriculotomy (2) Inducible VT on PES (2) Prior palliative shunt (2) QRS ≥ 180 msec (1) Bokma et al. [51] modification included CMR-assessed LV and RV function, excluded inducible VT on PES	*The annual risk of appropriate ICD therapy:* 0–2: low risk (0%) 3–5: intermediate risk (3.8%) 6–12: high risk (17.5%)	0.6 Bokma et al. [51] modification: 0.75
**Spanish ACHD** (**Oliver et al. [7]**, **2021**)	*Odds ratio in parentheses:* Lesion specific risk Low (3.4) Moderate (3.9) High (9.8) Age (0.98) Male sex (1.8) Syncope (4.1) Symptomatic ischemic heart disease (8) NSVT (5.3) QRS Duration (1.02) Moderate–severe LV or RV dysfunction (3.74) Moderate–severe systemic ventricular (3.75) or subpulmonary (2.72) hypertrophy	*Composite risk score for 5-year predicted risk of SCD:* Very low (<1%) Low (1–4%) Moderate (4–12%) High (>12%)	0.83
**PREVENTION-ACHD** (**Vehmeijer et al. [8]**, **2021**)	*7-point risk score:* CAD (1) NYHA II/III symptoms (1) SVT (1) LV impairment (1) RV impairment (1) QRS ≥ 120 msec (1) QT dispersion ≥ 70 msec (1)	*Annual risk of SCD:* 1–2: <1% 3: 1% 4: 3% 5: 6% 6: 14% 7: >25%	0.81
**Ghonim et al. [9]**, **2022**	*100-point risk score:* RV LGE extent (0–40) LV LGE presence (0–6) RV ejection fraction (4–10) LV ejection fraction (4–12) Peak VO_2_ uptake ≤ 17 mL/kg/min^2^ (0–6) BNP ≥ 127 ng/L (0–12) Sustained atrial arrhythmia (0–8) Age > 50 (0–6)	*Mortality/year:* 0–20: 0.2% 21–50: 0.7% ≥51: 4.4%	0.87

BNP, B natriuretic peptide; CMR, cardiac magnetic resonance; ICD, implantable cardioverter defibrillator; LGE, late gadolinium enhancement; LV, left ventricle; LVEDP, left ventricular end-diastolic pressure; NSVT, non-sustained ventricular tachycardia; NYHA, New York Heart Association class; PES, programmed electrical stimulation; RV, right ventricle; SCD, sudden cardiac death; VT, ventricular tachycardia.

## References

[1] Dobson R.J., Ramparsad N., Walker N.L., McConnachie A., Danton M.H.D. (2021). Outcomes of adults with repaired tetralogy of Fallot from the national Scottish Cohort. Cardiol. Young.

[2] James F.W., Kaplan S., Chou T.C. (1975). Unexpected cardiac arrest in patients after surgical correction of tetralogy of Fallot. Circulation.

[3] Quattlebaum T.G., Varghese J., A Neill C., Donahoo J.S. (1976). Sudden death among postoperative patients with tetralogy of Fallot: A follow-up study of 243 patients for an average of twelve years. Circulation.

[4] Dennis M., Moore B., Kotchetkova I., Pressley L., Cordina R., Celermajer D.S. (2017). Adults with repaired tetralogy: Low mortality but high morbidity up to middle age. Open Heart.

[5] Gatzoulis M.A., Balaji S., Webber S.A., Siu S.C., Hokanson J.S., Poile C., Rosenthal M., Nakazawa M., Moller J.H., Gillette P.C. (2000). Risk factors for arrhythmia and sudden cardiac death late after repair of tetralogy of Fallot: A multicentre study. Lancet.

[6] Khairy P., Silka M.J., Moore J.P., DiNardo J.A., Vehmeijer J.T., Sheppard M.N., van de Bruaene A., Chaix M.-A., Brida M., Moore B.M. (2022). Sudden cardiac death in congenital heart disease. Eur. Heart J..

[7] Oliver J.M., Gallego P., Gonzalez A.E., Avila P., Alonso A., Garcia-Hamilton D., Peinado R., Dos-Subirà L., Pijuan-Domenech A., Rueda J. (2021). Predicting sudden cardiac death in adults with congenital heart disease. Heart.

[8] Vehmeijer J.T., Koyak Z., Leerink J.M., Zwinderman A.H., Harris L., Peinado R., Oechslin E.N., Robbers-Visser D., Groenink M., Boekholdt S.M. (2021). Identification of patients at risk of sudden cardiac death in congenital heart disease: The PRospEctiVE study on implaNTable cardIOverter defibrillator therapy and suddeN cardiac death in Adults with Congenital Heart Disease (PREVENTION-ACHD). Heart Rhythm..

[9] Ghonim S., Gatzoulis M.A., Ernst S., Li W., Moon J.C., Smith G.C., Heng E.L., Keegan J., Ho S.Y., McCarthy K.P. (2022). Predicting Survival in Repaired Tetralogy of Fallot: A Lesion-Specific and Personalized Approach. JACC Cardiovasc. Imaging.

[10] Krieger E.V., Zeppenfeld K., DeWitt E.S., Duarte V.E., Egbe A.C., Haeffele C., Lin K.Y., Robinson M.R., Sillman C., Upadhyay S. (2022). Arrhythmias in Repaired Tetralogy of Fallot: A Scientific Statement From the American Heart Association. Circ. Arrhythmia Electrophysiol..

[11] Khairy P., Harris L., Landzberg M.J., Viswanathan S., Barlow A., Gatzoulis M.A., Fernandes S.M., Beauchesne L., Therrien J., Chetaille P. (2008). Implantable cardioverter-defibrillators in tetralogy of fallot. Circulation.

[12] Kapel G.F., Reichlin T., Wijnmaalen A.P., Piers S.R., Holman E.R., Tedrow U.B., Schalij M.J., Stevenson W.G., Zeppenfeld K. (2015). Re-Entry using anatomically determined isthmuses: A curable ventricular tachycardia in repaired congenital heart disease. Circ. Arrhythmia Electrophysiol..

[13] Kapel G.F., Sacher F., Dekkers O.M., Watanabe M., Blom N.A., Thambo J.-B., Derval N., Schalij M.J., Jalal Z., Wijnmaalen A.P. (2017). Arrhythmogenic anatomical isthmuses identified by electroanatomical mapping are the substrate for ventricular tachycardia in repaired tetralogy of Fallot. Eur. Heart J..

[14] Nakazawa M., Shinohara T., Sasaki A., Echigo S., Kado H., Niwa K., Oyama K., Yokota M., Iwamoto M. (2004). Arrhythmias late after repair of tetralogy of fallot—A Japanese Multicenter Study. Circ. J..

[15] Deanfield J.E., McKenna W.J., Hallidie-Smith K.A. (1980). Detection of late arrhythmia and conduction disturbance after correction of tetralogy of Fallot. Heart.

[16] Wu M.-H., Lu C.-W., Chen H.-C., Chiu S.-N., Kao F.-Y., Huang S.-K. (2015). Arrhythmic burdens in patients with tetralogy of Fallot: A national database study. Heart Rhythm..

[17] Fujita T., Yoshida A., Ichikawa M. (2022). A case report of paroxysmal complete atrioventricular block in a patient with dextrocardia and repaired tetralogy of Fallot. Eur. Heart J. Case Rep..

[18] Hokanson J.S., Moller J.H. (2001). Significance of early transient complete heart block as a predictor of sudden death late after operative correction of tetralogy of fallot. Am. J. Cardiol..

[19] Friedli B., Bolens M., Taktak M. (1988). Conduction disturbances after correction of tetralogy of Fallot: Are electrophysiologic studies of prognostic value?. J. Am. Coll. Cardiol..

[20] Diller G.-P., Kempny A., Alonso-Gonzalez R., Swan L., Uebing A., Li W., Babu-Narayan S., Wort S.J., Dimopoulos K., Gatzoulis M.A. (2015). Survival Prospects and Circumstances of Death in Contemporary Adult Congenital Heart Disease Patients Under Follow-Up at a Large Tertiary Centre. Circulation.

[21] Possner M., Tseng S.Y., Alahdab F., Bokma J.P., Lubert A.M., Khairy P., Murad M.H., Ben Ali W., Prokop L.J., Czosek R.J. (2020). Risk Factors for Mortality and Ventricular Tachycardia in Patients With Repaired Tetralogy of Fallot: A Systematic Review and Meta-analysis. Can. J. Cardiol..

[22] Geva T., Mulder B., Gauvreau K., Babu-Narayan S.V., Wald R.M., Hickey K., Powell A.J., Gatzoulis M.A., Valente A.M. (2018). Preoperative Predictors of Death and Sustained Ventricular Tachycardia after Pulmonary Valve Replacement in Patients With Repaired Tetralogy of Fallot Enrolled in the INDICATOR Cohort. Circulation.

[23] Atallah J., Corcia M.C.G., Walsh E.P. (2020). Ventricular Arrhythmia and Life-Threatening Events in Patients With Repaired Tetralogy of Fallot. Am. J. Cardiol..

[24] Ghai A., Silversides C., Harris L., Webb G.D., Siu S.C., Therrien J. (2002). Left ventricular dysfunction is a risk factor for sudden cardiac death in adults late after repair of tetralogy of fallot. J. Am. Coll. Cardiol..

[25] Khairy P., Landzberg M.J., Gatzoulis M.A., Lucron H., Lambert J., Marçon F., Alexander M.E., Walsh E.P. (2004). Value of programmed ventricular stimulation after tetralogy of fallot repair: A multicenter study. Circulation.

[26] Vehmeijer J.T., Koyak Z., Bokma J.P., Budts W., Harris L., Mulder B.J.M., de Groot J.R. (2018). Sudden cardiac death in adults with congenital heart disease: Does QRS-complex fragmentation discriminate in structurally abnormal hearts?. EP Eur..

[27] Müller J., Hager A., Diller G.-P., Derrick G., Buys R., Dubowy K.-O., Takken T., Orwat S., Inuzuka R., Vanhees L. (2015). Peak oxygen uptake, ventilatory efficiency and QRS-duration predict event free survival in patients late after surgical repair of tetralogy of Fallot. Int. J. Cardiol..

[28] Dietl C.A., Cazzaniga M.E., Dubner S.J., Pérez-Baliño N.A., Torres A.R., Favaloro R.G. (1994). Life-threatening arrhythmias and RV dysfunction after surgical repair of tetralogy of Fallot. Comparison between transventricular and transatrial approaches. Circulation.

[29] Valente A.M., Gauvreau K., Assenza G.E., Babu-Narayan S.V., Schreier J., Gatzoulis M.A., Groenink M., Inuzuka R., Kilner P.J., Koyak Z. (2014). Contemporary predictors of death and sustained ventricular tachycardia in patients with repaired tetralogy of Fallot enrolled in the INDICATOR cohort. Heart.

[30] Kapel G.F., Brouwer C., Jalal Z., Sacher F., Venlet J., Schalij M.J., Thambo J.-B., Jongbloed M.R., Blom N.A., de Riva M. (2018). Slow Conducting Electroanatomic Isthmuses: An Important Link Between QRS Duration and Ventricular Tachycardia in Tetralogy of Fallot. JACC Clin. Electrophysiol..

[31] Khairy P., Aboulhosn J., Gurvitz M.Z., Opotowsky A.R., Mongeon F.-P., Kay J.D., Valente A.M., Earing M.G., Lui G.K., Gersony D.R. (2010). Arrhythmia burden in adults with surgically repaired tetralogy of fallot: A multi-institutional study. Circulation.

[32] Das M.K., El Masry H. (2010). Fragmented QRS and other depolarization abnormalities as a predictor of mortality and sudden cardiac death. Curr. Opin. Cardiol..

[33] Bazoukis G., Garcia-Zamora S., Çinier G., Lee S., Gul E.E., Álvarez-García J., Miana G., Hayıroğlu M., Tse G., Liu T. (2022). Association of electrocardiographic markers with myocardial fibrosis as assessed by cardiac magnetic resonance in different clinical settings. World J. Cardiol..

[34] Bokma J.P., Winter M.M., Vehmeijer J.T., Vliegen H.W., van Dijk A.P., van Melle J.P., Meijboom F.J., Post M.C., Zwinderman A.H., Mulder B.J.M. (2017). QRS fragmentation is superior to QRS duration in predicting mortality in adults with tetralogy of Fallot. Heart.

[35] Egbe A.C., Miranda W.R., Mehra N., Ammash N.M., Missula V.R., Madhavan M., Deshmukh A.J., Abdelsamid M.F., Kothapalli S., Connolly H.M. (2018). Role of QRS Fragmentation for Risk Stratification in Adults With Tetralogy of Fallot. J. Am. Heart Assoc..

[36] On Y.K., Kim J.S., Park S.W., Yang J.-H., Jun T.-G., Kang I.-S., Lee H.J., Choe Y.H., Huh J. (2012). Relation of fragmented QRS complex to right ventricular fibrosis detected by late gadolinium enhancement cardiac magnetic resonance in adults with repaired tetralogy of fallot. Am. J. Cardiol..

[37] Waldmann V., Bouzeman A., Duthoit G., Koutbi L., Bessiere F., Labombarda F., Marquié C., Gourraud J.B., Mondoly P., Sellal J.M. (2020). Long-Term Follow-Up of Patients With Tetralogy of Fallot and Implantable Cardioverter Defibrillator: The DAI-T4F Nationwide Registry. Circulation.

[38] Diller G.-P., Kempny A., Liodakis E., Alonso-Gonzalez R., Inuzuka R., Uebing A., Orwat S., Dimopoulos K., Swan L., Li W. (2012). Left ventricular longitudinal function predicts life-threatening ventricular arrhythmia and death in adults with repaired tetralogy of fallot. Circulation.

[39] Hanneman K., Crean A.M., Wintersperger B.J., Thavendiranathan P., Nguyen E.T., Kayedpour C., Wald R.M. (2018). The relationship between cardiovascular magnetic resonance imaging measurement of extracellular volume fraction and clinical outcomes in adults with repaired tetralogy of Fallot. Eur. Heart J. Cardiovasc. Imaging.

[40] Moon T.J., Choueiter N., Geva T., Valente A.M., Gauvreau K., Harrild D.M. (2015). Relation of biventricular strain and dyssynchrony in repaired tetralogy of fallot measured by cardiac magnetic resonance to death and sustained ventricular tachycardia. Am. J. Cardiol..

[41] Ortega M., Triedman J.K., Geva T., Harrild D.M. (2011). Relation of left ventricular dyssynchrony measured by cardiac magnetic resonance tissue tracking in repaired tetralogy of fallot to ventricular tachycardia and death. Am. J. Cardiol..

[42] Orwat S., Diller G.-P., Kempny A., Radke R., Peters B., Kühne T., Boethig D., Gutberlet M., Dubowy K.-O., Beerbaum P. (2016). Myocardial deformation parameters predict outcome in patients with repaired tetralogy of Fallot. Heart.

[43] Ghonim S., Ernst S., Keegan J., Giannakidis A., Spadotto V., Voges I., Smith G.C., Boutsikou M., Montanaro C., Wong T. (2020). Three-Dimensional Late Gadolinium Enhancement Cardiovascular Magnetic Resonance Predicts Inducibility of Ventricular Tachycardia in Adults With Repaired Tetralogy of Fallot. Circ. Arrhythmia Electrophysiol..

[44] van Mil S., Heung T., Malecki S., Van L., Chang J., Breetvelt E., Wald R., Oechslin E., Silversides C., Bassett A.S. (2020). Impact of a 22q11.2 Microdeletion on Adult All-Cause Mortality in Tetralogy of Fallot Patients. Can. J. Cardiol..

[45] Calcagni G., Calvieri C., Baban A., Bianco F., Barracano R., Caputo M., Madrigali A., Kikina S.S., Perrone M.A., Digilio M.C. (2022). Syndromic and Non-Syndromic Patients with Repaired Tetralogy of Fallot: Does It Affect the Long-Term Outcome?. J. Clin. Med..

[46] Blais C., Burwash I.G., Mundigler G., Dumesnil J.G., Loho N., Rader F., Baumgartner H., Beanlands R.S., Chayer B., Kadem L. (2006). Projected valve area at normal flow rate improves the assessment of stenosis severity in patients with low-flow, low-gradient aortic stenosis: The multicenter TOPAS (Truly or Pseudo-Severe Aortic Stenosis) study. Circulation.

[47] Kauw D., Woudstra O.I., van Engelen K., Meijboom F.J., Mulder B.J., Schuuring M.J., Bouma B.J. (2020). 22q11.2 deletion syndrome is associated with increased mortality in adults with tetralogy of Fallot and pulmonary atresia with ventricular septal defect. Int. J. Cardiol..

[48] Koyak Z., de Groot J.R., Bouma B.J., Van Gelder I.C., Budts W., Zwinderman A.H., Mulder B.J. (2013). Symptomatic but not asymptomatic non-sustained ventricular tachycardia is associated with appropriate implantable cardioverter therapy in tetralogy of Fallot. Int. J. Cardiol..

[49] Bokma J.P., Geva T., Sleeper L.A., Lee J.H., Lu M., Sompolinsky T., Babu-Narayan S.V., Wald R.M., Mulder B.J., Valente A.M. (2023). Improved Outcomes after Pulmonary Valve Replacement in Repaired Tetralogy of Fallot. J. Am. Coll. Cardiol..

[50] Vehmeijer J.T., Koyak Z., Budts W., Harris L., Silversides C.K., Oechslin E.N., Bouma B.J., Zwinderman A.H., Mulder B.J., de Groot J.R. (2017). Prevention of Sudden Cardiac Death in Adults with Congenital Heart Disease: Do the Guidelines Fall Short?. Circ. Arrhythmia Electrophysiol..

[51] Bokma J.P., de Wilde K.C., Vliegen H.W., van Dijk A.P., van Melle J.P., Meijboom F.J., Zwinderman A.H., Groenink M., Mulder B.J.M., Bouma B.J. (2017). Value of Cardiovascular Magnetic Resonance Imaging in Noninvasive Risk Stratification in Tetralogy of Fallot. JAMA Cardiol..

[52] Kakarla J., Denham N.C., Ishikita A., Oechslin E., Alonso-Gonzalez R., Nair K. (2023). Risk Stratification for Sudden Cardiac Death in Repaired Tetralogy of Fallot. CJC Pediatr. Congenit. Heart Dis..

[53] Ishikita A., McIntosh C., Hanneman K., Lee M.M., Liang T., Karur G.R., Roche S.L., Hickey E., Geva T., Barron D.J. (2023). Machine Learning for Prediction of Adverse Cardiovascular Events in Adults With Repaired Tetralogy of Fallot Using Clinical and Cardiovascular Magnetic Resonance Imaging Variables. Circ. Cardiovasc. Imaging.

[54] Tandon A., Nguyen H.H., Avula S., Seshadri D.R., Patel A., Fares M., Baloglu O., Amdani S., Jafari R., Inan O.T. (2023). Wearable Biosensors in Congenital Heart Disease: Needs to Advance the Field. JACC Adv..

[55] Sandhu A., Ruckdeschel E., Sauer W.H., Collins K.K., Kay J.D., Khanna A., Jaggers J., Campbell D., Mitchell M., Nguyen D.T. (2018). Perioperative electrophysiology study in patients with tetralogy of Fallot undergoing pulmonary valve replacement will identify those at high risk of subsequent ventricular tachycardia. Heart Rhythm..

[56] Stout K.K., Daniels C.J., Aboulhosn J.A., Bozkurt B., Broberg C.S., Colman J.M., Crumb S.R., Dearani J.A., Fuller S., Gurvitz M. (2019). 2018 AHA/ACC Guideline for the Management of Adults With Congenital Heart Disease: Executive Summary: A Report of the American College of Cardiology/American Heart Association Task Force on Clinical Practice Guidelines. J. Am. Coll. Cardiol..

[57] Wald R.M., Valente A.M., Marelli A. (2015). Heart failure in adult congenital heart disease: Emerging concepts with a focus on tetralogy of Fallot. Trends Cardiovasc. Med..

[58] Babu-Narayan S.V., Uebing A., Davlouros P.A., Kemp M., Davidson S., Dimopoulos K., Bayne S., Pennell D.J., Gibson D.G., Flather M. (2012). Randomised trial of ramipril in repaired tetralogy of Fallot and pulmonary regurgitation: The APPROPRIATE study (Ace inhibitors for Potential PRevention of the deleterious effects of Pulmonary Regurgitation in Adults with repaired TEtralogy of Fallot). Int. J. Cardiol..

[59] Bokma J.P., Winter M.M., van Dijk A.P., Vliegen H.W., van Melle J.P., Meijboom F.J., Post M.C., Berbee J.K., Boekholdt S.M., Groenink M. (2018). Effect of Losartan on Right Ventricular Dysfunction: Results From the Double-Blind, Randomized REDEFINE Trial (Right Ventricular Dysfunction in Tetralogy of Fallot: Inhibition of the Renin-Angiotensin-Aldosterone System) in Adults With Repaired Tetralogy. Circulation.

[60] Norozi K., Bahlmann J., Raab B., Alpers V., Arnhold J.O., Kuehne T., Klimes K., Zoege M., Geyer S., Wessel A. (2007). A prospective, randomized, double-blind, placebo controlled trial of beta-blockade in patients who have undergone surgical correction of tetralogy of Fallot. Cardiol. Young.

[61] Thambo J.-B., De Guillebon M., Xhaet O., Dos Santos P., Roubertie F., Labrousse L., Iriart X., Ploux S., Haissaguerre M., Bordachar P. (2013). Biventricular pacing in patients with Tetralogy of Fallot: Non-invasive epicardial mapping and clinical impact. Int. J. Cardiol..

[62] Furushima H., Chinushi M., Sugiura H., Komura S., Tanabe Y., Watanabe H., Washizuka T., Aizawa Y. (2006). Ventricular tachycardia late after repair of congenital heart disease: Efficacy of combination therapy with radiofrequency catheter ablation and class III antiarrhythmic agents and long-term outcome. J. Electrocardiol..

[63] Henmi R., Ejima K., Yagishita D., Iwanami Y., Nishimura T., Takeuchi D., Toyohara K., Shoda M., Hagiwara N. (2017). Long-Term Efficacy of Implantable Cardioverter Defibrillator in Repaired Tetralogy of Fallot—Role of Anti-tachycardia Pacing. Circ. J..

[64] Waldmann V., Bouzeman A., Duthoit G., Koutbi L., Labombarda F., Gourraud J.-B., Mondoly P., Sellal J.M., Bordachar P., Hermida A. (2022). Sex Differences in Outcomes of Tetralogy of Fallot Patients With Implantable Cardioverter-Defibrillators. JACC Clin. Electrophysiol..

[65] Egbe A.C., Miranda W.R., Madhavan M., Ammash N.M., Missula V.R., Al-Otaibi M., Fatola A., Kothapalli S., Connolly H.M. (2019). Cardiac implantable electronic devices in adults with tetralogy of Fallot. Heart.

[66] Willy K., Reinke F., Bögeholz N., Köbe J., Eckardt L., Frommeyer G. (2019). The entirely subcutaneous ICDTM system in patients with congenital heart disease: Experience from a large single-centre analysis. EP Eur..

[67] D’souza B.A., Epstein A.E., Garcia F.C., Kim Y.Y., Agarwal S.C., Belott P.H., Burke M.C., Leon A.R., Morgan J.M., Patton K.K. (2016). Outcomes in Patients With Congenital Heart Disease Receiving the Subcutaneous Implantable-Cardioverter Defibrillator: Results From a Pooled Analysis From the IDE Study and the EFFORTLESS S-ICD Registry. JACC Clin. Electrophysiol..

[68] Alonso P., Osca J., Cano O., Pimenta P., Andrés A., Yagüe J., Millet J., Rueda J., Sancho-Tello M.J. (2017). The Role of Conventional and Right-Sided ECG Screening for Subcutaneous ICD in a Tetralogy of Fallot Population. Pacing Clin. Electrophysiol..

[69] Koyak Z., de Groot J.R., Van Gelder I.C., Bouma B.J., van Dessel P.F., Budts W., van Erven L., van Dijk A.P., Wilde A.A., Pieper P.G. (2012). Implantable cardioverter defibrillator therapy in adults with congenital heart disease: Who is at risk of shocks?. Circ. Arrhythmia Electrophysiol..

[70] (2020). L.Goldenthal, I.; Rosenbaum, M.S.; Lewis, M.; Sciacca, R.R.; Garan, H.; Biviano, A.B. Inappropriate implantable cardioverter-defibrillator shocks in repaired tetralogy of fallot patients: Prevalence and electrophysiological mechanisms. IJC Heart Vasc..

[71] Kawada S., Chakraborty P., Downar E., Sanchez A.-P., Sathananthan G., Bhaskaran A., Kugamoorthy P., Albertini L., Oechslin E.N., Silversides C. (2021). The Role of Ablation in Prevention of Recurrent Implantable Cardioverter Defibrillator Shocks in Patients With Tetralogy of Fallot. CJC Open.

[72] Laredo M., Frank R., Waintraub X., Gandjbakhch E., Iserin L., Hascoët S., Himbert C., Gallais Y., Hidden-Lucet F., Duthoit G. (2017). Ten-year outcomes of monomorphic ventricular tachycardia catheter ablation in repaired tetralogy of Fallot. Arch. Cardiovasc. Dis..

[73] van Zyl M., Kapa S., Padmanabhan D., Chen F.C., Mulpuru S.K., Packer D.L., Munger T.M., Asirvatham S.J., McLeod C.J. (2016). Mechanism and outcomes of catheter ablation for ventricular tachycardia in adults with repaired congenital heart disease. Heart Rhythm..

[74] Moore J.P., Seki A., Shannon K.M., Mandapati R., Tung R., Fishbein M.C. (2013). Characterization of anatomic ventricular tachycardia isthmus pathology after surgical repair of tetralogy of fallot. Circ. Arrhythmia Electrophysiol..

[75] Laredo M., Duthoit G., Sacher F., Anselme F., Audinet C., Bessière F., Bordachar P., Bouzeman A., Boveda S., Bun S.S. (2023). Rapid ventricular tachycardia in patients with tetralogy of Fallot and implantable cardioverter-defibrillator: Insights from the DAI-T4F nationwide registry. Heart Rhythm..

[76] Baumgartner H., De Backer J., Babu-Narayan S.V., Budts W., Chessa M., Diller G.-P., Lung B., Kluin J., Lang I.M., Meijboom F. (2021). 2020 ESC Guidelines for the management of adult congenital heart disease. Eur. Heart J..

[77] Therrien J., Siu S.C., McLaughlin P.R., Liu P.P., Williams W.G., Webb G.D. (2000). Pulmonary valve replacement in adults late after repair of tetralogy of fallot: Are we operating too late?. J. Am. Coll. Cardiol..

[78] Bessière F., Gardey K., Bouzeman A., Duthoit G., Koutbi L., Labombarda F., Marquié C., Gourraud J.B., Mondoly P., Sellal J.M. (2021). Impact of Pulmonary Valve Replacement on Ventricular Arrhythmias in Patients With Tetralogy of Fallot and Implantable Cardioverter-Defibrillator. JACC Clin. Electrophysiol..

[79] Bokma J.P., Geva T., A Sleeper L., Narayan S.V.B., Wald R., Hickey K., Jansen K., Wassall R., Lu M., A Gatzoulis M. (2018). A propensity score-adjusted analysis of clinical outcomes after pulmonary valve replacement in tetralogy of Fallot. Heart.

[80] Verzaal N.J., Massé S., Downar E., Nanthakumar K., Delhaas T., Prinzen F.W. (2021). Exploring the cause of conduction delays in patients with repaired Tetralogy of Fallot. EP Eur..

[81] Therrien J., Siu S.C., Harris L., Dore A., Niwa K., Janousek J., Williams W.G., Webb G., Gatzoulis M.A. (2001). Impact of pulmonary valve replacement on arrhythmia propensity late after repair of tetralogy of fallot. Circulation.

[82] Bouyer B., Jalal Z., Ramirez F.D., Derval N., Iriart X., Duchateau J., Roubertie F., Tafer N., Tixier R., Pambrun T. (2023). Electrophysiological study prior to planned pulmonary valve replacement in patients with repaired tetralogy of Fallot. J. Cardiovasc. Electrophysiol..

[83] Waldmann V., Bessière F., Gardey K., Bakloul M., Belli E., Bonnet D., Chaussade A.-S., Cohen S., Delasnerie H., Dib N. (2023). Systematic Electrophysiological Study Prior to Pulmonary Valve Replacement in Tetralogy of Fallot: A Prospective Multicenter Study. Circ. Arrhythmia Electrophysiol..

